# Hydrogen-rich water irrigation enhances fruit quality in ‘Flame Seedless’ grapes by regulating chlorophyll fluorescence parameters and antioxidant activities

**DOI:** 10.3389/fpls.2025.1693075

**Published:** 2025-10-28

**Authors:** Hossam Salah Mahmoud Ali, Huanhuan Zhang, Dongdong Yao, Liu Kun, Fengyun Zhao, Jianrong Feng, Kun Yu

**Affiliations:** ^1^ Department of Horticulture, College of Agriculture, Shihezi University, Shihezi, Xinjiang, China; ^2^ Key Laboratory of Special Fruits and Vegetables Cultivation Physiology and Germplasm Resources Utilization of Xinjiang Production and Construction Corps, Department of Horticulture, College of Agriculture, Shihezi University, Shihezi, Xinjiang, China; ^3^ Department of Horticulture, College of Agriculture, Minia University, El-Minya, Egypt

**Keywords:** hydrogen-rich water, antioxidant enzymes, chlorophyll fluorescence, Flame Seedless grapes, fruit quality, reactive oxygen species, anthocyanin accumulation

## Abstract

**Introduction:**

Hydrogen-rich water (HRW) plays a crucial role in regulating plant growth and development. However, its potential involvement in modulating photosynthetic pigments, chlorophyll fluorescence (ChlF) parameters, antioxidant enzyme activities, and fruit ripening in *(Vitis vinifera L.)* ‘Flame Seedless’ grapes grown in a greenhouse remain unclear.

**Methods:**

This study aimed to investigate the effects of HRW irrigation at a concentration of 1.0 mg L^-1^ on chlorophyll pigments, ChlF parameters, enzymatic antioxidant activities, and fruit quality.

**Results:**

HRW irrigation induced a significant increase in carotenoid (Car) content, which was observed only on the first day after irrigation. It also significantly enhanced chlorophyll a (Chl a) and chlorophyll b (Chl b) contents, as well as ChlF parameters such as maximum quantum efficiency of photosystem II (Fv/Fm), and the photochemical quantum yield of PSII (ΦPSII), while non-photochemical quenching (NPQ) decreased, indicating enhanced PSII functionality and photosynthetic performance. Antioxidant enzyme activities, including superoxide dismutase (SOD), peroxidase (POD), and catalase (CAT), were also enhanced, reducing reactive oxygen species (ROS) accumulation and maintaining ROS homeostasis in grapevine leaves. HRW treatment promoted the accumulation of secondary metabolites such as total phenolic content (TPC), total flavonoid content (TFC), total anthocyanin content (TAC), and Car, which contributed to an improved color index of red grapes (CIRG). Additionally, fruit quality was improved by increasing total soluble solids (TSS), soluble sugars, and pH, while reducing fruit firmness and titratable acidity (TA). Berry weight and overall yield were also enhanced compared with control plants.

**Discussion:**

These results demonstrate that HRW is a promising and sustainable approach for enhancing photosynthetic performance, antioxidant defense, secondary metabolite accumulation, and fruit quality in greenhouse-grown ‘Flame Seedless’ grapes, providing a practical basis for improving grape cultivation and production.

## 1 Introduction

Hydrogen gas (H_2_) is a sustainable energy source that reduces carbon emissions and promotes green energy (Kamshybayeva et al., 2024). H_2_, the most abundant element in the universe, is a small, and colorless, odorless, and tasteless gas capable of easily diffusing through cell membranes (Russell et al., 2020; Yu et al., 2023; Li et al., 2022a). It participates in atmospheric redox reactions, mainly from methane oxidation and volatile organics (Zgonnik, 2020). Produced by bacteria, algae, and plants via hydrogenases and nitrogenases (Li et al., 2022a), H_2_ is also released by rhizobia during nitrogen fixation (Xu et al., 2023). Although H_2_ treatments in crops have been shown to improve both yield and quality, its gaseous form presents challenges for practical application (Russell et al., 2020; Hu et al., 2021). Being lighter than air, H_2_ is difficult to retain under field conditions, making direct gaseous application impractical. Additionally, its high flammability limits its use at significant concentrations due to safety and storage concerns. A more feasible method involves using saturated forms of H_2_, such as HRW. Which can be directly applied to plants either through foliar spraying or soil drenching with water as demonstrated in previous (Wu et al., 2020b). HRW is commonly prepared using several techniques. One involves dissolving magnesium-based tablets in water; however, this approach may introduce residual by-products into the solution. Alternatively, HRW can be generated by bubbling or pumping H_2_ gas into distilled water or other irrigation media using hydrogen generators. The prepared solution can then be diluted to the desired concentration for treatment (Zulfiqar et al., 2021). HRW suppresses ROS by lowering redox potential, creating a more reducing environment that favors electron donation and directly scavenges radicals such as hydrogen peroxide (H_2_O_2_) and superoxide anions (O2^•-^), while simultaneously enhancing antioxidant enzyme activities and modulating gene expression to restore cellular redox balance. This integrates redox-driven reactions with enzymatic and genetic defenses, thereby mitigating oxidative stress and enhancing plant resilience (Franceschelli et al., 2016; Qazi, 2022; Ding et al., 2024a). Due to its unique climatic conditions and light-heat resources, Xinjiang Province has contributed significantly to grape culture development in China, accounting for 19% of the planting area and 24% of total grape output nationally (Cheng et al., 2021). Although Xinjiang remains a prominent agricultural region due to its sunlight-rich environment and advanced irrigation practices, sustaining long-term crop productivity and fruit quality under its intensive cultivation systems necessitates a shift toward more physiologically informed strategies (Wang et al., 2018; Han et al., 2024).

Because these antioxidant mechanisms are closely linked to photosynthetic stability, it becomes essential to monitor the functional performance of the photosynthetic apparatus under fluctuating conditions. In this context, the measurement of ChlF is a non-invasive technique used to assess the activity of photosystem II (PSII), offering valuable insights into plant responses to environmental changes, photosynthetic mechanisms, and growth (Pashkovskiy et al., 2018). This method involves high-frequency measurements using a fluorometer, where dark-adapted leaves are exposed to brief pulses of actinic light (Rochaix, 2011). The resulting fluorescence kinetics provide crucial information on parameters such as Fv/Fm, ΦPSII, the photochemical quenching coefficient (qP), the electron transport rate (ETR), and NPQ, which are fundamental for assessing light utilization efficiency in photosynthesis (Chen et al., 2021; Mvondo-She et al., 2024). These physiological indicators not only reflect the plant’s ability to cope with oxidative stress but also serve as a basis for evaluating the efficacy of agronomic interventions aimed at enhancing plant resilience. In this regard, as plants become increasingly vulnerable to both biotic and abiotic stresses, researchers are turning toward more environmentally sustainable approaches to improve productivity and fruit quality without exacerbating ecological burdens.

Under normal conditions, ROS are byproducts of essential metabolic processes, including respiration, photosynthesis, and photorespiration. However, under stress conditions, molecules such as O2^•-^ and •H_2_O_2_ are overproduced (Yun et al., 2021). Excessive ROS accumulation, particularly under environmental stress or during senescence, can cause oxidative damage (Ali et al., 2024). To mitigate the adverse effects of these reactive molecules, plants activate specialized enzymatic defense systems that safeguard cellular function, including POD, SOD, CAT, and polyphenol oxidase (PPO), which regulate redox homeostasis and are crucial for delaying senescence and maintaining cellular integrity (Karimi et al., 2022; Guo et al., 2025).

Accordingly, grapevine (*Vitis vinifera*) has garnered particular attention due to its economic importance as a horticultural crop, its sensitivity to environmental stressors, and its deep-rooted association with human civilization. This species belongs to the Vitaceae family and includes two main subspecies, depending on their mode of reproduction and whether they are cultivated or wild: the wild grapevine (*Vitis vinifera ssp. sylvestris*), which continues to thrive in woodland ecosystems extending from Western Europe and North Africa to regions near the Himalayas, and the domesticated (*Vitis vinifera ssp. vinifera, or sativa*), which originated along river valleys across North America, Europe, and Asia. It is an adaptable fruit shrub that grows in hot tropical, subtropical, and temperate climates, and in a variety of soil types (Rodriguez-Izquierdo et al., 2024). Grapes rank second globally among fruit crops in terms of cultivated area and yield, following citrus (Estrada et al., 2021). They are cultivated on approximately 7.1 million hectares, yielding about 28.39 million metric tons annually, with China ranking third globally, managing around 753,000 hectares of vineyards in 2024 (OIV, 2025). Northwestern China, with long sunshine hours, sufficient light, and large diurnal temperature differences, is an excellent production area for grapes (Liang et al., 2014; Liu et al., 2025).

Within this context, ‘Flame Seedless’ has emerged as one of the most prominent and widely cultivated table grape cultivars. It is highly popular among consumers and is recognized for its seedlessness, early ripening, compact clusters, uniform berry size, and attractive bright red coloration, which is further enhanced by its elevated TAC. Moreover, it is noted for its crisp peel, juicy pulp, and distinctive flavor (Lo’ay and El-Boray, 2018; Abdel-Sattar et al., 2022). Despite these desirable characteristics, sustaining optimal productivity and fruit quality necessitates a thorough understanding of its physiological and biochemical requirements. ‘Flame Seedless’ is particularly sensitive to environmental factors such as light intensity, water availability, and temperature, all of which substantially influence photosynthetic efficiency, antioxidant activity system, and expression of defense-related genes (Lecourieux et al., 2017; Chen et al., 2022; Pei et al., 2023).

Although greenhouse cultivation provides a relatively controlled environment, microclimatic fluctuations, high planting density, and other cultivation-related stresses can still compromise photosynthetic performance, redox homeostasis, pigment stability, and overall fruit quality. Consequently, interventions that enhance intrinsic physiological and biochemical resilience are valuable even under greenhouse conditions. HRW has been demonstrated to regulate antioxidant enzyme activities, maintain ROS homeostasis, stabilize chlorophyll and PSII function, and optimize energy metabolism (Yu et al., 2023; Ferrandino et al., 2023; Dinis et al., 2024; Yu et al., 2024; Akbar et al., 2025).Moreover, HRW application can improve preharvest fruit attributes, including firmness, TSS, soluble sugars, and coloration, while reducing TA and promoting accumulation of secondary metabolites, thereby mitigating postharvest deterioration (Dong et al., 2023; Yao et al., 2024).

Despite extensive reports on HRW efficacy in laboratory and open-field conditions, its impact on greenhouse-grown ‘Flame Seedless’ grapevines remain inadequately characterized. Evaluating HRW under greenhouse conditions is essential to elucidate its potential as a practical agronomic strategy for enhancing physiological performance and fruit quality within relatively controlled cultivation systems. Accordingly, this study aims to investigate the effects of preharvest HRW application via subsurface drip irrigation on antioxidant enzyme activities, ChlF, and fruit quality of greenhouse-grown ‘Flame Seedless’ grapevines.

## 2 Materials and methods

### 2.1 Plant material and treatments

The grape ‘Flame Seedless’ was treated in the greenhouse at the Shihezi University Base, Xinjiang, China (45°20′N, 86°03′E) from June 2023 to August 2024. To investigate the effects of HRW on grape fruit quality, ChlF, enzymatic antioxidants, and microbial communities, the treatments were arranged as follows:

1. HRW treatment at a concentration of 1.0 mg L^-1^ according to previous studies (Li et al., 2022b).
2. Control (water without adding H_2_ gas).

**Figure 1:**
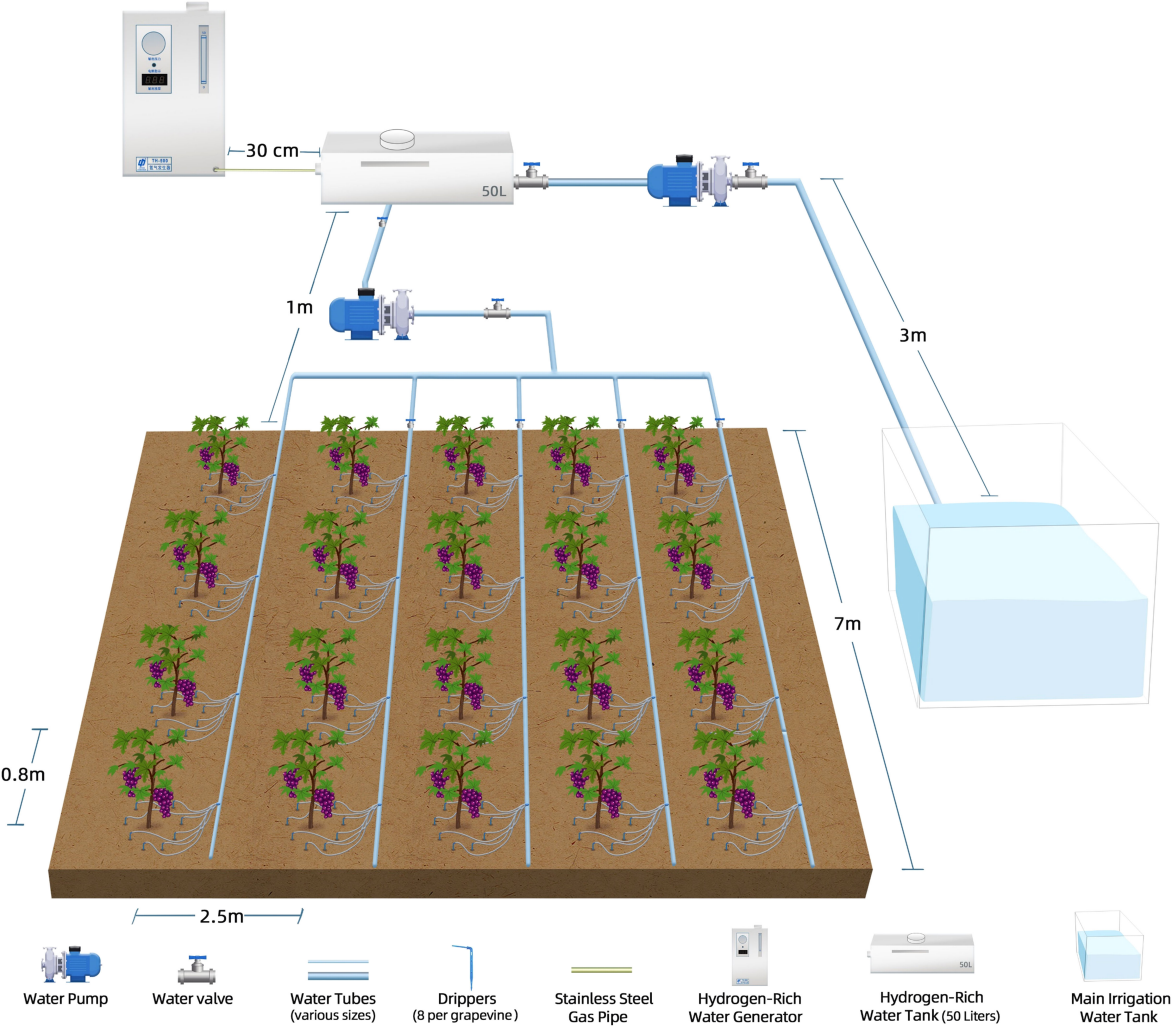
Schematic diagram of the experimental setup. The irrigation system includes a main irrigation water tank, a water pump, and a series of water tubes of various sizes connected to water valves and drippers (4per grape seedlings). The HRWgeneration and delivery system consists of a HRWgenerator, a stainless steel air pipe for gas transfer, and a 50-liter HRW tank. The system ensures the precise delivery of HRW to the grape seedlings through a subsurface irrigation network designed to optimize water distribution and efficiency.

The greenhouse soil was sandy, with the following basic physical and chemical properties: pH 7.21, organic matter content 13.67 g·kg^-1^, total nitrogen 0.38 g·kg^-1^, rapidly available phosphorus 25.6 mg·kg^-1^, and rapidly available potassium 21.3 mg·kg^-1^. Grapevines were planted in 2018, and the vineyard was organized with vertical trellises oriented east and the grape rows in a north-south direction. Cement upright pillars were erected at two ends of each grape tree row. Four galvanized iron wires were laid on the pillars, and the trellis was approximately 1.5 m. Each row contained five grape plants with a row spacing of 2.5 m and a plant spacing of 0.8 m. During winter pruning, six fertile branches were maintained on each grape tree. All grape plants were irrigated and fertilized with Hoagland nutrient solution at 1 g/L (pH 5.8, 25°C; Coolaber Technology Co., Ltd., Beijing, China) through an underground drip irrigation system. HRW was produced using a generator (HT-500; Beijing Zhonghuipu Analytical Technology Research Institute, Beijing, China). High-purity H_2_ gas (99.999% [v/v]) from the generator was infused into a 50 L plastic tank for 4 hours at a flow rate of 500 mL min^-1^. The container was completely filled and tightly sealed to prevent H_2_ loss and to enhance its dissolution in water. The concentration of dissolved H_2_ was approximately 1.0 mg L^-1^, measured using a portable dissolved H_2_ meter (ENH-2000; TRUSTLEX, Osaka, Japan) calibrated via gas chromatography. The residence time of H2 in the above water was more than 12 h. Once produced, HRW was piped to the trees for subsurface drip irrigation (flow rate: 4 L/h, eight drippers per grapevine) (**Figure 1**). HRW or water (control) was applied via subsurface drip irrigation every three days. Each grapevine received 10 liters of solution per irrigation event, delivered through the drippers at a constant flow rate. This irrigation frequency and volume were maintained consistently throughout the treatment period from June 30 (30 days after flowering) to August 10, 2024. Each treatment included six biological replicates, with one grapevine per replicate, uniformly selected based on similar growth status. Measurements were taken at four key intervals: 44, 54, 64, and 74 days after full bloom (DB44, DB54, DB64, and DB74, respectively) to estimate fruit quality traits. Additionally, measurements were conducted on the 1st, 4th, and 7th days of the same irrigation cycle (DI1, DI4, and DI7, respectively) to assess ChlF parameters and chlorophyll pigments in leaves. On the final day of the experiment (August 10), cluster and berry weights, and leaf antioxidant enzyme activity.

### 2.2 Determination of chlorophyll pigments and Car in peel and leaves

Pigment contents were determined using the methods of Li et al. (2024c). Berry peels were crushed, and fresh leaves (veins removed) were cut into small pieces. In each case, 0.1 g of sample was extracted with 10 mL of 95% ethanol in the dark for 24 hours. After centrifugation (12,000 ×g for 10 min),the absorbance of the supernatant was measured at 665, 649, and 470 nm using a UV spectrophotometer. Pigment contents were calculated based on absorbance readings, solution volume, and sample mass according to the following (Equations 1–5):


(1) $$\begin{array}{c} \text{Chl a }\left(\text{mg}\cdot {\text{g}}^{-1}\text{fw}\right)\\ =\left[\left(13.36*{\text{A}}_{665}\right)-\left(5.19*{\text{A}}_{649}\right)\right]*[\left(\text{V}/\left(1000*\text{W}\right)\right] \end{array}$$

(2) $$\begin{array}{c} \text{Chl b }\left(\text{mg}\cdot {\text{g}}^{-1}\text{fw}\right)\\ =[\left(27.43*{\text{A}}_{649}\right)-\left(8.12*{\text{A}}_{665}\right)*[\left(\text{V}/\left(1000*\text{W}\right)\right] \end{array}$$

(3) $$\begin{array}{c} \text{Total chlorophyll content }\left(\text{TChl}\right)\;\left({\text{mg}\cdot \text{gn}}^{1}\text{ fw}\right)\\ =\text{ Chl a}+\text{Chl b} \end{array}$$

(4) Chlorophyll a/b (Chl a/b)=(Chl a)/(Chl b)

(5) $$\begin{array}{c} \text{Car }\left(\text{mg}\cdot {\text{g}}^{1}\text{ fw}\right)\\ =[\left(100*{\text{A}}_{470}\right)+\left(76.438*{\text{A}}_{665}\right)\\ -\left(266.721*{\text{A}}_{649}\right)]*[\;\left(\text{V}/\left(209000*\text{W}\right)\right] \end{array}$$

Where A665, A649, and A470 represent the absorbance values of the extracted solution at wavelengths of 665 nm, 649 nm, and 470 nm, respectively; V is the volume of the solvent mixture (mL); and W is the weight of the sample (g).

### 2.3 Leaf ChlF measurements

The measurement of ChlF was conducted on the 4th to 6th fully expanded leaf of each plant using a pulse-amplitude-modulation fluorometer (IMAG-CG, Walz, Germany). Leaves were dark-adapted in a humid environment for 30 minutes before measurement. Parameters recorded included: F_0_ (minimum fluorescence, dark-adapted), F′_0_ (minimum fluorescence, light-adapted), Fm (maximum fluorescence, dark-adapted), F′m (maximum fluorescence, light-adapted), and Ft (steady-state fluorescence under illumination) (Abdullaev et al., 2024). These values were used for subsequent calculations (González-Salvatierra et al., 2024) (Equations 6–10):


(6) Fv/Fm=(Fm−F0)/Fm

(7) ΦPSII=(F'm−Ft)/(F'm)

(8) NPQ=(F0−F'm)/(F'm)

(9) qP =(F'm−Ft)/(F'm−F'0)

(10) ETR=ΦPSII *PPFD*0.84*0.5

PPFD is the photon flux density recorded by the leaf clip sensor. 0.84 represents the proportion of light absorbed by the photosystems, and 0.5 accounts for photon distribution between them.

### 2.4 Determination of antioxidant enzymes activity, H_2_O_2_, and O_2_^•-^

The activity of antioxidant enzymes in the leaves, such as POD, SOD, PPO, and CAT, were measured using an assay kit (http://www.cominbio.com/index.html; Catalog Nos. POD-2-Y, SOD-2-W, PPO-2-Y, and CAT-2-Y) (Ngaffo Mekontso et al., 2021). Assay kits (http://geruisi-bio.com; Catalog Nos. G0116W for O_2_^•^-^^ and G0168W for H_2_O_2_) were used to determine the O_2_^•-^ and H_2_O_2_ content (Ruan et al., 2024). In brief, ‘Flame seedless’ grapevine leaves were removed from −80°C and immediately ground finely in liquid nitrogen. The frozen powder was weighed (0.1 g) and homogenized in 1 ml of the extraction solution. The extracts were centrifuged at 8,000 × g for 10 min at 4°C. The supernatants were collected and used for the determination of ROS and enzyme activities. The absorbance of H_2_O_2_, O_2_^•-^, SOD, POD, CAT, APX and PPO were measured by a UV spectrophotometer at 510, 540, 560, 470, 240, 290, and 525 nm, respectively. All enzymes activity and ROS were measured according to the manufacturer’s instructions, and the results were expressed as specified in these instructions.

### 2.5 Determination of TAC, TPC, and TFC in fruit peels

The extraction of TAC, TPC, and TFC compounds was carried out using HCl-methanol (1:99, v/v) following the method described by Xu et al. (2021) with some modifications. Peel samples were homogenized on ice, subjected to ultrasonic extraction for 30 minutes in the dark, stored at 4°C for 15 hours, and centrifuged at 8,000 × g for 3 minutes at 4°C. The tissue-to-buffer ratio was 1:40 (w/v). TAC was quantified using the pH differential spectrophotometric method Da Porto and Natolino (2018) with modifications. Absorbance was measured at 520 nm and 700 nm in buffer solutions at pH 1.0 (KCl-HCl) and pH 4.5 (NaAc-HCl) after incubating diluted samples (1 mL sample + 9 mL buffer) at room temperature for 1 hour. Results were expressed as mg cyanidin-3-glucoside per kg fresh weight (mg cyd-glu·kg^-1^ FW) (Equations 11, 12):


(11) $$\text{ TAC}(\text{mg}.{\text{kg}}^{-1}\text{ FW})=\left(\text{A}*\text{MW}*\text{DF}\right)/\left(\text{ϵ}*\text{W}\right)$$

(12) $$\text{A}=({\text{A}}_{502}-{\text{A}}_{700}){\text{ pH}}^{1.0}-(A_{520}-A_{700}){\text{ pH}}^{4.5}$$

MW is the molecular mass of cyanidin 3-O-glucoside (449.2), ϵ is its molar extinction coefficient (26,900 mol^-1^), Df is the dilution factor, and W is the sample’s fresh weight.

TFC was determined using a colorimetric assay (Xie et al., 2015). Sample extracts and a rutin standard solution were diluted (1:4, v/v) to 5 mL with distilled water in a 10 mL flask. Then, 0.3 mL of 5% NaNO_2_ was added and shaken for 5 minutes, followed by 0.3 mL of 10% AlCl_3_ with stirring for 6 minutes. Subsequently, 2 mL of 1 M NaOH and 2.4 mL of distilled water were added and mixed thoroughly. TFC was calculated using a rutin calibration curve (0–200 mg/L) and expressed as mg rutin equivalent (RE) per 100 g fresh weight (mg RE/100 g FW). The absorbance was determined at 510 nm. TPC was determined using the modified Folin–Ciocalteu method (Vasco et al., 2008). Briefly, 0.5 mL of extract, blank, or standard solution was mixed with 0.5 mL of Folin–Ciocalteu reagent in a 25 mL volumetric flask and stirred for 3 minutes. Then, 10 mL of 75 g/L Na_2_CO_3_ solution was added, homogenized, and diluted to 25 mL with distilled water. The mixture was incubated at room temperature for 1 hour, and absorbance was measured at 750 nm using a Shimadzu UV-160A spectrophotometer. Results were expressed as gallic acid equivalents (GAE) based on a standard curve (0–240 mg/L).

### 2.6 Determination of fruit quality-related characteristics

#### 2.6.1 Berry and cluster weights and yield assessment

At the maturity stage (August 10, 2024), three grapevines per treatment were randomly selected. Cluster weights were measured using a precision balance (accuracy: 0.01 g). Fifty berries were randomly sampled from the upper, middle, and lower parts of clusters to determine single berry weight, with three replicates. Yield per acre (tons) was estimated by multiplying the average cluster weight, the number of clusters per vine, and the number of vines per acre based on the experimental planting density.

#### 2.6.2 Fruit firmness and color

Fruit firmness was measured using a fruit firmness tester (GY-B, Quzhou Aipu, Co., Ltd., China) with at least three replicates (Wang et al., 2024). Berry peel color was assessed with a Minolta CR-200 colorimeter (Konica Minolta, Japan), recording L* (lightness), a* (red, green), and b* (yellow, blue) coordinates. Measurements were taken from the equator of 30 randomly selected berries, including top, middle, and bottom samples. The CIRG was calculated based on the chromaticity values (Zhou et al., 2024). The calculation formula for the CIRG is as follows (Equations 13–15):


(13) $$\text{CIRG }=\left(180-\text{h}\right)/\left({\text{L}}^{*}+{\text{C}}^{*}\right)$$

(14) $$c^{*}=\sqrt{\left({\left(\text{a*}\right)}^{2}+{\left(\text{b*}\right)}^{2}\right)}$$

(15) $$\text{h }(0-360)=tan(b^{*})/(a^{*})$$

Where:

C* = Chroma, representing color intensity

h° = Hue angle, representing the perceived color

#### 2.6.3 TSS, pH, and TA

Grape juice was extracted by manually pressing and filtering 15–30 grapes per treatment for analysis of TA, TSS, and pH. TSS was measured using a digital refractometer (PAL-1, ATAGO, Japan) with 1 mL of the sample, following the manufacturer’s instructions (Hu et al., 2025). pH was measured with a digital pH meter (PH818, Hanoi, Vietnam), calibrated with pH 4.0 and 6.8 standard solutions (AiHaiti et al., 2025). TA was determined by titration with 0.1 M NaOH to a pH endpoint of 8.1, using phenolphthalein as the indicator, and expressed as tartaric acid equivalents per 100 g fresh berry weight, calculated using the following (Equation 16):


(16) TA%=(VI×N×VII×f)/(W×VIII) ×100 

VI is the total extract volume (mL), N is the NaOH concentration (mol/L), VII is the NaOH volume used (mL), VIII is the extract volume used (mL), W is the sample weight (g), and *f* is the acid factor (0.075 for tartaric acid).

#### 2.6.4 Total soluble sugar

Total soluble sugar was determined using the anthrone colorimetry method (Kanwal et al., 2024) with modifications. Fresh fruit tissue (1.0 g) was boiled in 10 mL distilled water for 60 minutes, filtered into a 100 mL flask, and the process was repeated to recover residues, adjusting the volume to 100 mL. A 0.5 mL extract was mixed with 1.5 mL distilled water, 0.5 mL anthrone reagent, and 5.0 mL concentrated sulfuric acid, incubated at 100°C for 1 minute, cooled, and absorbance measured at 630 nm. Glucose was used to construct the standard curve (0–100 µg/mL).

### 2.7 Statistical analysis

Experimental data are presented as mean ± standard deviation (SD) (n = 3) and were analyzed using GraphPad Prism version 8.02 software (GraphPad Software, San Diego, CA, USA). A multiple Student’s t-test was employed to determine significant differences between the control and treatment groups (*P< 0.05, **P< 0.01, ***P< 0.001).

## 3 Results

### 3.1 Effect of HRW treatment on berry and cluster weights

The HRW treatment influenced berry fresh weight, cluster weight, and estimated yield of ‘Flame Seedless’ grapes at different stages. Berry fresh weight increased progressively under HRW treatment, reaching the maximum value of 1.973 g at DB74, which represented an increase of 7.64% compared with the control (**Table 1**). This increase in berry weight was reflected in the cluster weight, with HRW-treated clusters averaging 591.67 g at DB74, compared with 551.00 g in control, corresponding to an increase of 7.38%. Similarly, the estimated yield per acre was higher in HRW-treated vines, reaching 9.94 t compared with 9.26 t in the control at DB74, indicating an increase of 7.38% (**Table 1**). The HRW treatment therefore consistently enhanced individual berry weight, cluster weight, and overall yield throughout the fruit development period.

**Table 1:** Effect of irrigation with HRW on berry weight, cluster weight, and yield of “Flame seedless” grapes.

Treatment	Berry weight (g)	Cluster weight (g)	Estimated yield (Ton. acre^−1^)
HRW	1.97 ± 0.05a	591.66 ± 8.50a	9.94 ± 0.14a
H2O	1.83 ± 0.05b	551 ± 18.40b	9.26 ± 0.31b

Values are presented as means + SD (n = 3). Different letters within a row indicate signiﬁcant differences by the student’s t test among treatments (p< 0.05).

### 3.2 Effect of HRW treatment on photosynthetic pigments and ChlF parameters in the leaves of ‘Flame Seedless’ grapes

**Figure 2:**
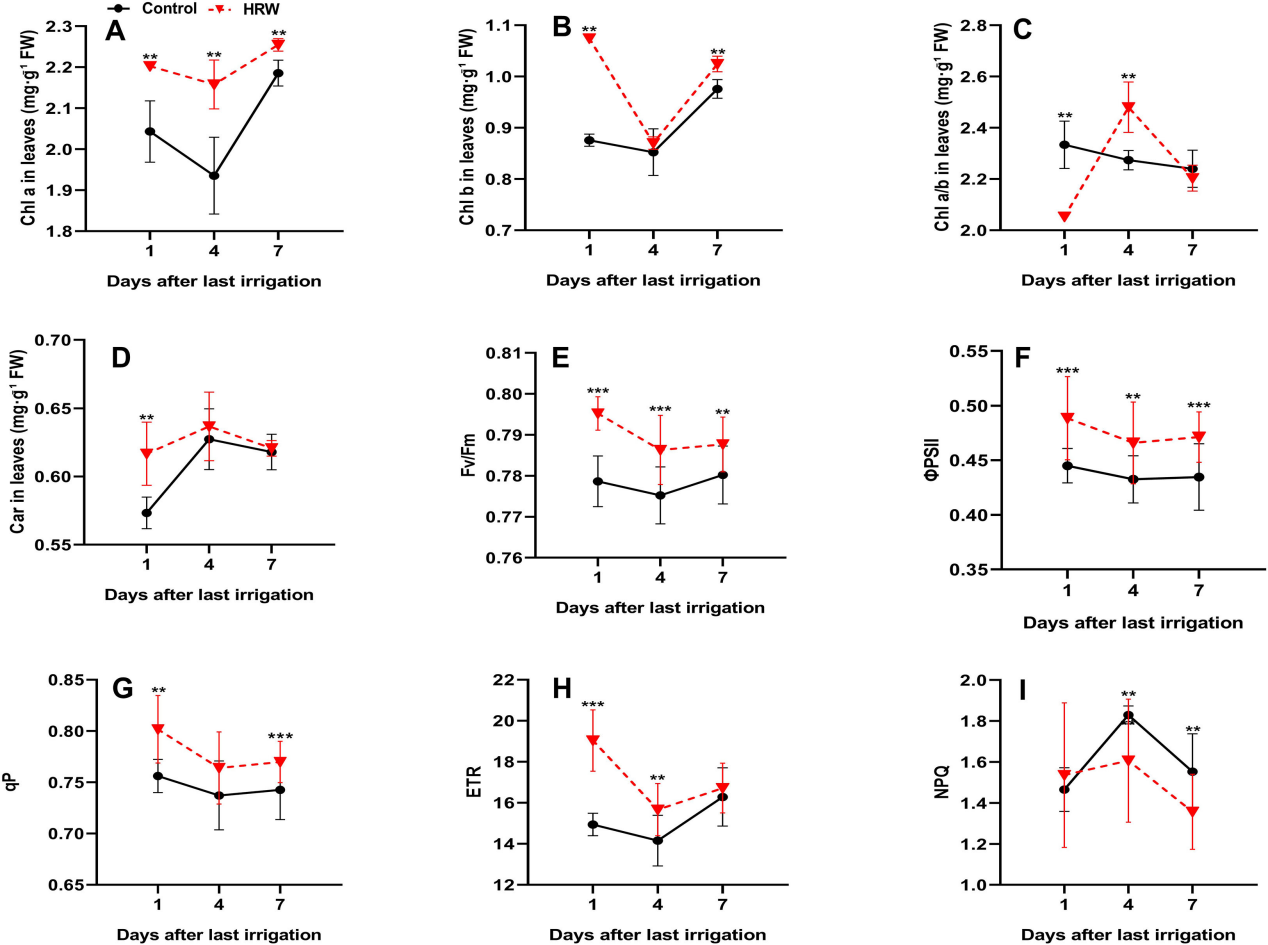
Effects of HRW treatment on chlorophyll a content (Chl a) **(A)**, chlorophyll b content (Chl b) **(B)**, chlorophyll a/b ratio (Chl a/b) **(C)**, carotenoid content (Car) **(D)**, maximal quantum yield of PSII (Fv/Fm) **(E)**, photochemical quantum yield of PSII (ΦPSII) **(F)**, photochemical quenching coefficient (qP) **(G)**, electron transport rate (ETR) **(H)**, and non-photochemical quenching (NPQ) **(I)** in ‘Flame Seedless’ grape leaves. Values (means ± SD) were from three replicates. Asterisks indicate significant differences from the control (Student’s t-test; *P< 0.05, **P< 0.01, ***P< 0.001).

HRW treatment markedly influenced both photosynthetic pigments and ChlF parameters, reflecting enhanced PSII functionality and photosynthetic performance. As shown in **Figure 2**, Chl a content in HRW-treated leaves was always significantly higher than in the control (P< 0.05), with increases of 7.80%, 11.49%, and 3.16% at DI1, DI4, and DI7, respectively (**Figure 2A**). Similarly, Chl b also increased significantly under HRW treatment at DI1 and DI7 by 22.69% and 4.99%, respectively, compared with the control (P< 0.05), while no significant difference was detected at DI4 (**Figure 2B**). In contrast, the Chl a/b ratio exhibited a fluctuating trend. At DI1, HRW-treated leaves showed a significantly lower ratio than the control, with a reduction of 12.14%, whereas a significant increase of 9.23% was recorded at DI4. By DI7, only a slight change was observed, and the difference between treatments was not significant (**Figure 2C**). For Car content in HRW-treated leaves increased significantly at DI1 by 7.81% compared with the control (P< 0.05). The content continued to rise, reaching the highest value at DI4 for both treatments, although the difference from the control at this stage was not statistically significant. By DI7, Car levels slightly decreased compared with DI4, with a minimal and nonsignificant difference between HRW and control leaves (0.49%) (**Figure 2D**). These results indicate that HRW promoted Car accumulation, particularly enhancing light-harvesting potential during the early stages, with peak levels occurring at DI4, even though the difference at this point was not statistically significant. Fv/Fm in HRW-treated leaves exhibited an increase of 2.13% at DI1 (P< 0.05). By DI4, a decline was observed in both treatments; however, HRW-treated leaves retained a statistically significant enhancement of 1.43% relative to the control (P< 0.05). At DI7, Fv/Fm values increased slightly in both treatments, with HRW remaining 0.95% higher than the control, although the difference was not significant (**Figure 2E**). Correspondingly, ΦPSII demonstrated consistent and significant improvement under HRW, with increments of 9.76% at DI1, 7.71% at DI4, and 8.41% at DI7 (P< 0.05) (**Figure 2F**). This pattern was complemented by qP, which increased by 6.02% at DI1 (P< 0.05). At DI4, qP declined in both treatments. By DI7, qP rose modestly, with HRW-treated leaves exhibiting a 3.65% enhancement over the control (P< 0.05) (**Figure 2G**). In parallel, ETR values showed significant increases in HRW-treated leaves, being 27.41% higher than the control at DI1 (P< 0.05). On DI4, ETR decreased in both treatments, yet HRW still displayed a 10.68% improvement over the control (P< 0.05). At DI7, ETR increased moderately, with HRW exceeding the control, although the difference was not statistically significant (**Figure 2H**). In contrast, NPQ exhibited a progressive reduction under HRW, with decreases of 0.49% at DI1 (non-significant), 4.82% at DI4 (P< 0.05), and 12.59% at DI7 (P< 0.05) (**Figure 2I**).

### 3.3 Impact of HRW treatment on enzyme activities and ROS metabolism in ‘Flame Seedless’ grape leaves

**Figure 3:**
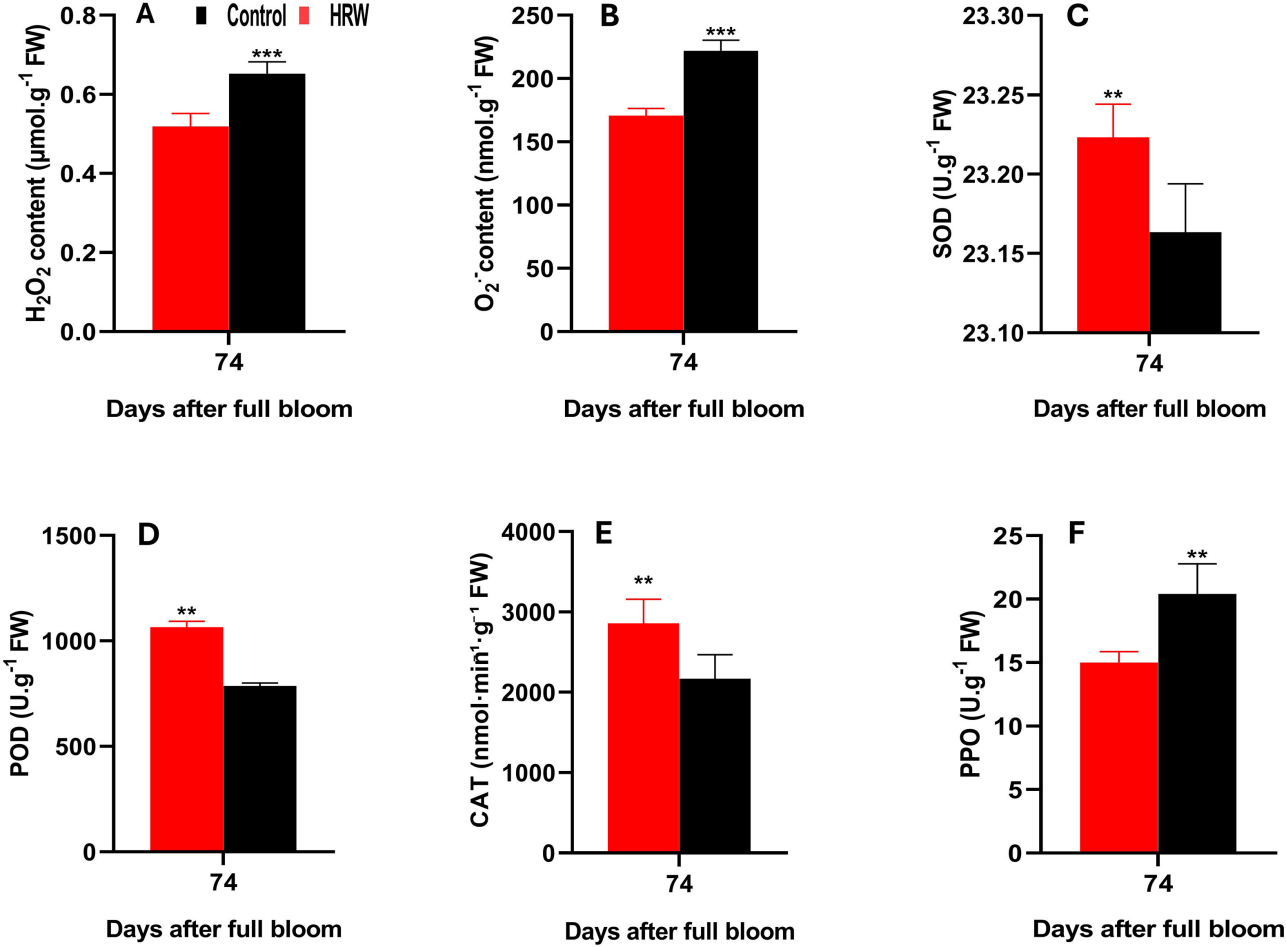
Effects of HRW treatment on hydrogen peroxide (H_2_O_2_) content **(A)**, superoxide anion (O_2_˙^-^) content **(B)**, and the activities of superoxide dismutase (SOD) **(C)**, peroxidase (POD) **(D)**, catalase (CAT) **(E)**, and polyphenol oxidase (PPO) **(F)** in ‘Flame Seedless’ grape leaves. Data are presented as means ± SD from three replicates. Significant differences from the control were determined by Student’s t-test (**P< 0.01, ***P< 0.001).

As shown in **Figure 3**, HRW treatment markedly influenced ROS accumulation in grape leaves. The content of H_2_O_2_ in HRW-treated leaves was 0.519 μmol·g^-1^ FW, representing a significant reduction of 20.5% compared with the control, which recorded 0.652 μmol·g^-1^ FW (P< 0.05) (**Figure 3A**). Similarly, O_2_·^-^ levels were lower under HRW treatment, with an average of 170.81 nmol·g^-1^ FW, in contrast to 222.06 nmol·g^-1^ FW in the control, corresponding to a decrease of 23.1% (P< 0.05) (**Figure 3B**). In contrast, HRW treatment significantly enhanced the activities of key antioxidant enzymes, including SOD, POD, and CAT (P< 0.05). The average activity of SOD in HRW-treated leaves reached 28.84 U·g^-1^ FW, compared with 25.18 U·g^-1^ FW in control (**Figure 3C**). Similarly, POD activity was markedly higher under HRW treatment, with a mean value of 1065.33 U·g^-1^ FW, whereas the control showed only 786.67 U·g^-1^ FW (**Figure 3D**). CAT activity also exhibited a significant elevation, averaging 2857.5 nmol·min^-1^·g^-1^ FW under HRW treatment, in contrast to 2168.2 nmol·min^-1^·g^-1^ FW in the control (**Figure 3E**). Notably, PPO activity was significantly reduced in HRW-treated leaves, with a mean value of 15.00 U·g^-1^ FW, compared to 20.40 U·g^-1^ FW in the control (**Figure 3F**).

### 3.4 Effect of HRW treatment on TChl and Car in berry peels

**Figure 4:**
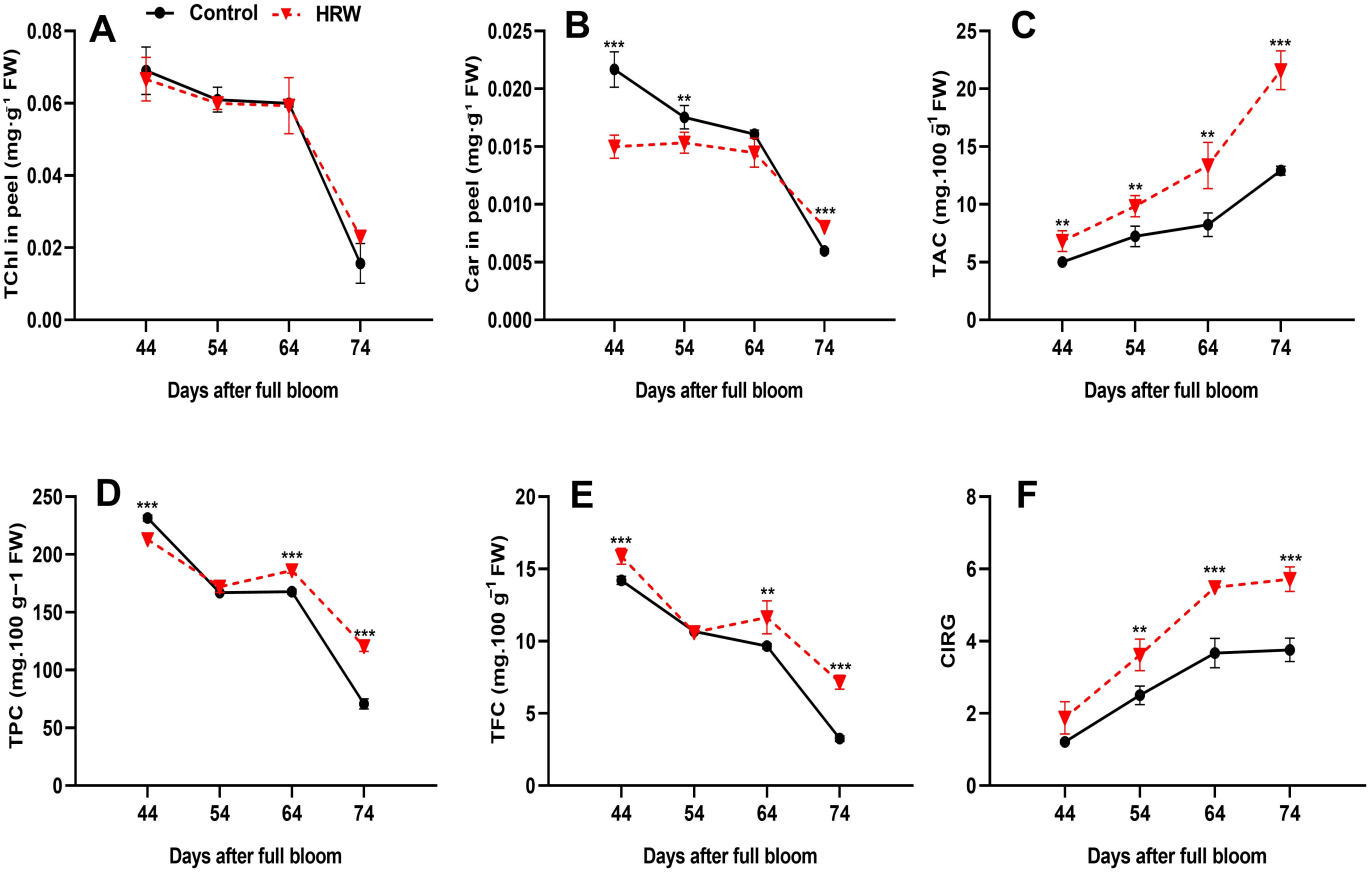
Effects of HRW treatment on total chlorophyll content (TChl) **(A)**, carotenoid content (Car) **(B)**, total anthocyanin content (mg cyanidin 3-O-glucoside 100 g^-1^ FW) (TAC) **(C)**, total phenolic content (mg GAE 100 g^-1^ FW) (TPC) **(D)**, total flavonoid content (mg QE 100 g^-1^ FW) (TFC) **(E)**, and the color index of red grapes (CIRG) **(F)** in ‘Flame Seedless’ grape peel. Data are presented as means ± SD from three replicates. Significant differences from the control were determined by Student’s t-test (**P< 0.01, ***P< 0.001).

A rapid decrease in TChl content in the berry peel was observed during fruit ripening in both treatments (**Figure 4A**). Compared to the control, HRW treatment reduced the TChl in berry peel at the DB44, DB54, and DB64 stages; however, the differences were not statistically significant. Notably, the control treatment showed significantly higher Car levels than HRW at DB44 and DB54, with increases of 30.77% and 12.55% (P< 0.05), respectively. By DB64, no significant differences were observed. At DB74, HRW maintained higher Car levels than the control, corresponding to an increase of 34.64% (P< 0.05) (**Figure 4B**).

### 3.5 HRW improved the TAC, TPC, TFC and CIRG in berry peels

In the berry peels, TAC in both treatments showed a gradually increasing trend as the fruit matured. At all stages, the TAC levels with HRW were significantly higher than those in the control group, with values of 9.85, 13.36, and 21.60 mg 100 g^-1^ FW at DB54, DB64, and DB74, respectively (P< 0.05) (**Figure 4C**). Anthocyanins, phenolic compounds, and flavonoids are closely related antioxidant secondary metabolites, with anthocyanins considered a subclass of flavonoids. All these compounds share biosynthetic pathways and compete for common metabolic precursors, which suggest that HRW may exert a synergistic effect on their coordinated accumulation. To further understand the source of this antioxidant activity, changes in TPC and TFC were measured. The results showed that. TPC content decreased progressively from DB44 to DB74. However, TPC content was higher under HRW treatment than in the control at DB54, DB64, and DB74, with values of 172.00, 186.13, and 120.53 mg 100 g^-1^ FW, respectively, while it was lower at DB44 with a value of 213.07 mg 100 g^-1^ FW (**Figure 4D**). Similarly, at all stages except DB54, HRW treatment led to higher TFC content compared with the control, with values of 11.65 mg 100 g^-1^ FW at DB64 and 7.16 mg 100 g^-1^ FW at DB74, while it was slightly lower at DB54 with a value of 10.60 mg 100 g^-1^ FW (**Figure 4E**). Notably, both TPC and TFC content initially decreased to DB54 before rising again, though they followed an overall descending trend up to DB74. While TPC and TFC exhibited a descending trend, CIRG values increased consistently during fruit ripening (**Figure 4F**). At all-time points, CIRG values were higher under HRW treatment than in the control, with values of 3.62, 5.49, and 5.717 at DB54, DB64, and DB74, respectively (P< 0.05).

### 3.6 Effect of HRW treatment on fruit firmness, TA, TSS, soluble sugar content, and pH in berry fruits

**Figure 5:**
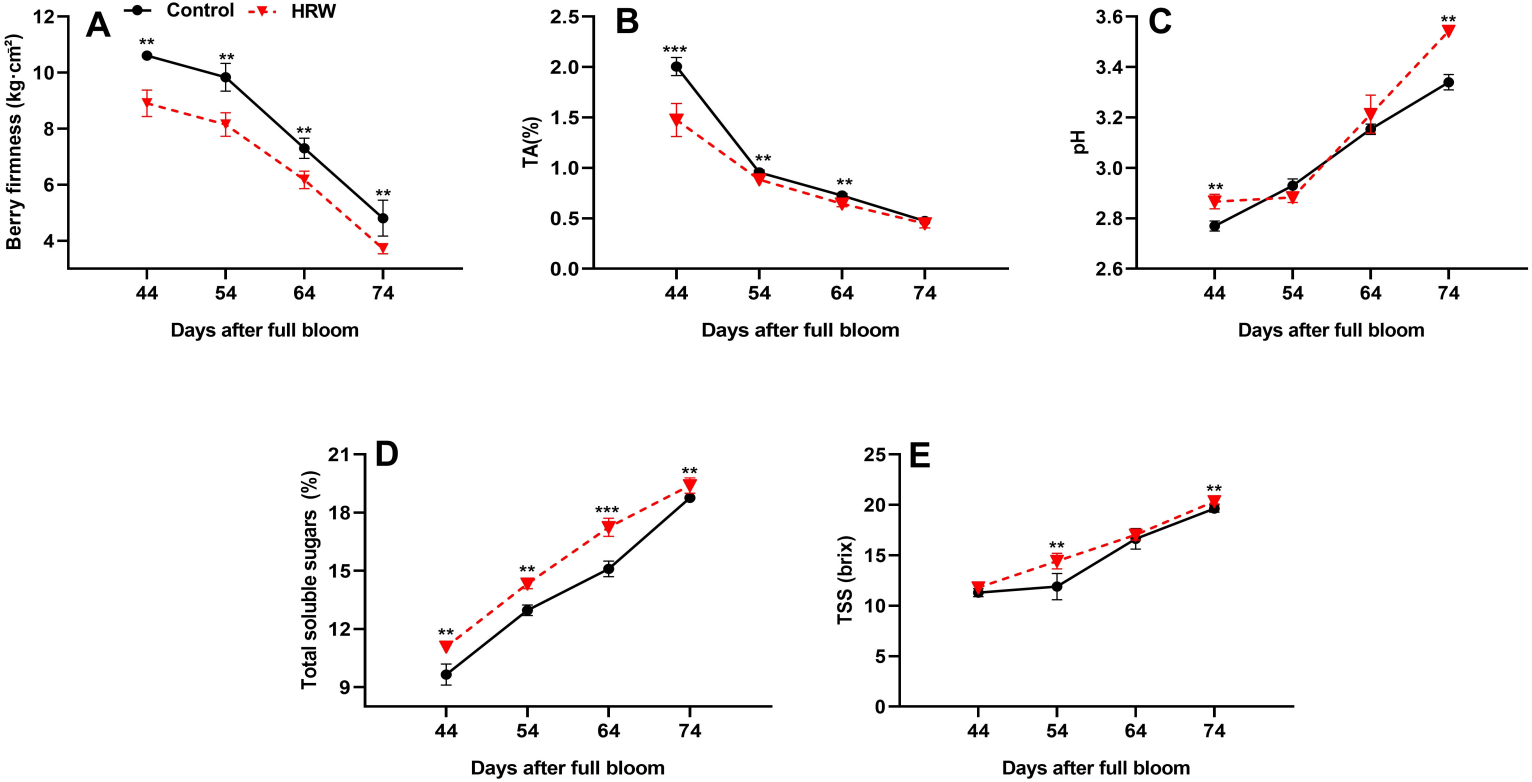
Effects of HRW treatment on fruit firmness **(A)**, titratable acidity (TA) **(B)**, pH **(C)**, total soluble sugar **(D)**, and total soluble solids (TSS) **(E)** in the berries of ‘Flame Seedless’ grapes. Values (means ± standard deviations) were calculated from three independent replicates. Asterisks indicate significant differences compared to the control (Student’s t-test; **P< 0.01, ***P< 0.001).

The quality attributes of ‘Flame Seedless’ grapes were significantly influenced by HRW treatment compared to control. This influence is shown in **Figure 5**; the firmness and TA of grape fruits showed a gradually decreasing trend as the fruits matured. At DB74, fruit firmness and TA reached their lowest values, with HRW-treated grapes showing a 22.75% and 5.47%, respectively, reduction compared to the control (**Figures 5A, B**). Conversely, the TSS, soluble sugar content, and pH of grape fruits in each treatment gradually increased as the fruits matured. At DB54 and DB74, the content of TSS in the HRW treatment was significantly higher than control (P< 0.05), with an increase of 21.18% and 3.65%, respectively (**Figure 5C**). The soluble sugar content also showed a consistent rise as the fruit matured, with HRW treatment outperforming control treatment at DB44, DB54, DB64, and DB74 by 14.85%, 10.54%, 14.19%, and 3.41%, respectively (P< 0.05), and this sugar accumulation reflects improved fruit quality (**Figure 5D**). On the other hand, the pH of grape fruits in both treatments was not significantly different at DB54 and DB64, but at DB44 and DB74, the pH of the HRW treatment was significantly higher than the control (P< 0.05), with an increase of 3.49% and 6.09%, respectively (**Figure 5E**).

## 4 Discussion

### 4.1 HRW treatment improves yield of ‘Flame Seedless’ fruit

ChlF is a critical determinant of photosynthetic product accumulation, while the photosynthetic rate directly reflects the plant’s photosynthetic capacity (Xiong et al., 2020). Enhanced photosynthetic efficiency accelerates biomass accumulation, ultimately influencing fruit yield. In this study, HRW treatment significantly increased single berry weight, cluster weight, and estimated total yield compared with the control (**Table 1**). These observations are consistent with prior reports demonstrating that higher photosynthetic pigment content, improved photosynthetic parameters, and overall photosynthetic performance positively affect final plant yield (Zhang et al., 2023b).

Moreover, the observed positive correlation between HRW treatment and antioxidant enzyme activities in leaves suggests that enhanced ROS scavenging and physiological stability contribute to improved photosynthetic efficiency. Collectively, these findings indicate that HRW application promotes both fruit development and overall yield formation in ‘Flame Seedless’ grapes, highlighting its potential for improving production under greenhouse cultivation.

### 4.2 Impact of HRW on photosynthetic pigments, ChlF, ROS homeostasis, and antioxidant enzyme activity in ‘Flame Seedless’ grape leaves

Photosynthetic pigments and ChlF parameters are essential indicators of photosynthetic efficiency in plants. Chlorophyll, the primary pigment responsible for converting light energy into chemical energy, also serves as a key marker of both physiological and ecological status (Zhang et al., 2021; Xu et al., 2022b). In our study, control plants exhibited the lowest levels of both Chl a and Chl b at DI4, while HRW-treated plants maintained higher levels across all time points, particularly at DI4 (**Figures 2A, B**). The lower chlorophyll pigment levels in control plants may reflect impaired chloroplast integrity and enhanced degradation of photosynthetic pigments under fluctuating soil moisture conditions. These findings highlight the potential effect of HRW on chlorophyll stability and photosynthetic efficiency, likely due to enhanced antioxidant activity and delayed chlorophyll breakdown (El-Saadony et al., 2024; Liao et al., 2025; Wang et al., 2025).

Notably, The Chl a/b ratio and Car increased at DI4 in both treatments, with HRW-treated plants showing the highest values (**Figures 2C, D**). This increase likely represents a photoprotective adaptation, as Car dissipate excess light energy and quench singlet oxygen, thereby stabilizing thylakoid membranes (Zgonnik, 2020; Abdel-Sattar et al., 2024; He et al., 2024; Rao et al., 2025). A higher Chl a/b ratio may also indicate selective retention of Chl a to support PSII activity under fluctuating soil moisture conditions (Xu et al., 2022b). These pigment dynamics suggest that HRW mitigates chlorophyll degradation and enhances photoprotection by regulating gene expression involved in pigment biosynthesis, thereby protecting chlorophyll while promoting Car accumulation (Zhao et al., 2020; Wang et al., 2025).

Photosynthesis relies on the absorption of light energy by light-harvesting complexes to initiate photochemical reactions (Akhlaq et al., 2025). The efficiency of PSII can be evaluated using ChlF parameters such as ΦPSII, qP, ETR, Fv/Fm, and NPQ, which are highly responsive to environmental changes, and provide insights into the functional state of the photosynthetic machinery (Abdullaev et al., 2024). Among these parameters, Fv/Fm declined at DI4 and recovered at DI7, with HRW-treated plants consistently exhibiting higher values than controls (**Figure 2E**). This trend indicates reduced photoinhibition and enhanced light-use efficiency, in agreement with previous findings in tomato (Hazrati et al., 2016; Akhlaq et al., 2025). Similarly, ΦPSII and qP—indicators of light energy utilization efficiency and photochemical quenching—decreased significantly at DI4 and partially recovered by DI7 after the second irrigation cycle. HRW-treated plants consistently exhibited higher values than the control group (**Figures 2F, G**), suggesting enhanced electron transport and improved PSII stability (Wang et al., 2022; Abdullaev et al., 2024). ETR also declined at DI4, reflecting inhibition of the electron transport chain due to soil moisture fluctuations. However, HRW-treated plants exhibited significantly higher ETR values, indicating better protection of PSII and more stable electron flow. By DI7, ETR values recovered in both groups, with HRW-treated plants maintaining superior performance. In contrast, NPQ, representing non-photochemical quenching or the thermal dissipation of excess excitation energy, increased significantly at DI4, reflecting a photoprotective mechanism (**Figure 2I**). Similar observations have been reported in Aloe vera, where NPQ rose under fluctuating water availability (Wang et al., 2022). Elevated NPQ is generally associated with proton accumulation in the thylakoid lumen due to transient saturation of the electron transport chain (Hazrati et al., 2016). Notably, HRW-treated plants exhibited lower NPQ levels, indicating a reduced requirement for thermal dissipation and more efficient regulation of photosynthetic activity.

The enhanced photosynthetic performance in HRW-treated plants is associated with the activation of strong antioxidant defenses that protect chloroplasts from oxidative stress. As the primary sites of photosynthesis, chloroplasts are highly sensitive to changes in the surrounding environment, which can disrupt CO_2_ assimilation, leading to over-reduction of the ETR and excessive ROS generation (El-Saadony et al., 2024). Accumulated ROS can impair PSII, damage thylakoid membranes, and degrade pigments, reducing photochemical efficiency as reflected by Fv/Fm and related ChlF parameters (Shah et al., 2021; Iwai et al., 2023; Rao et al., 2025). Plants mitigate such oxidative damage through a complex antioxidant system comprising both enzymatic and non-enzymatic components (Li et al., 2024b).

HRW has been shown to support redox balance by enhancing antioxidant enzyme activity and mitigating ROS levels. It directly reduces O_2_˙^-^ content, forming H_2_O_2_, which is subsequently scavenged through enhanced H_2_O_2_-degrading activity (Yun et al., 2021). In the present study, ROS levels in grapevine leaves decreased markedly under HRW treatment compared with the control (**Figure 3**), consistent with similar reductions reported in litchi (Yun et al., 2021) and pakchoi (An et al., 2021). This decline in ROS suggests the involvement of antioxidant enzyme systems, which serve as the primary defense against oxidative stress in plant tissues and play a central role in protecting cellular structures (Wei et al., 2019).

Specifically, SOD catalyzes the dismutation of O_2_˙^-^ into H_2_O_2_ and O_2_, while CAT and POD further degrade H_2_O_2_ into H_2_O and O_2_, preventing the harmful accumulation of ROS (Wei et al., 2019; Li et al., 2024b; Guo et al., 2025). In this study, HRW treatment significantly enhanced the activities of SOD, POD, and CAT in grapevine leaves (**Figures 2C–E**). Comparable responses have been observed in Hypsizygus marmoreus mushrooms (Ali et al., 2024), pakchoi (An et al., 2021), red pitaya (Ding et al., 2024a), and Chinese cabbage (Li et al., 2022a). Conversely, HRW suppressed PPO activity in grapevine leaves (**Figure 2F**), consistent with results reported in Lanzhou Lily (Zhang et al., 2023a).

The beneficial effects of HRW on antioxidant enzyme activity and ROS scavenging may be linked to its modulation of gene expression related to oxidative stress regulation (Yang et al., 2024). HRW enhances the expression of the transcription factor HcMYB6, which upregulates genes encoding antioxidant enzymes, thereby improving ROS scavenging capacity (Wei et al., 2019; Yun et al., 2021; Ding et al., 2024a, 2024b). In contrast, HRW suppresses the expression of the transcriptional repressor HcR2R3MYB, which is associated with PPO activity, thus reducing enzymatic browning and preserving phenolic compounds (Ding et al., 2024a). In addition to enzymatic defenses, non-enzymatic antioxidants such as Car, TFC, and TPC contribute to ROS scavenging, stabilization of thylakoid membranes, and protection of pigment–protein complexes (Hasanuzzaman et al., 2020). Car, in particular, dissipate excess excitation energy and prevent triplet chlorophyll formation, thereby reducing photoinhibition-related ROS (Hasanuzzaman et al., 2020). Collectively, these dual regulatory effects of HRW—strengthening both enzymatic and non-enzymatic antioxidant systems—enhance the physiological integrity of leaves and fruits, maintain their quality, and support plant performance under different environmental conditions surrounding the plant.

Our results suggest that HRW application enhances both enzymatic and non-enzymatic antioxidant responses, upregulating the expression and activity of SOD, POD, CAT, and PPO while also increasing pools of Car. These biochemical improvements correspond with observed increases in Fv/Fm, ΦPSII, qP, ETR, and a reduction in NPQ, all indicating enhanced photochemical efficiency. In addition, HRW support mitochondrial ATP synthesis, reinforcing the energy supply required for photosynthetic carbon metabolism under natural fluctuations soil water availability between irrigation cycles (He et al., 2024).Furthermore, HRW-treated plants maintained optimal Chl a/b ratios (~2.2), which are essential for efficient light harvesting and energy transfer within the photosystems. This stability is likely linked to the preservation of chloroplast ultrastructure and the efficient operation of their intrinsic antioxidant machinery, which maintains redox homeostasis and prevents oxidative damage under these conditions; such protection supports sustained photosynthetic functionality, as previously reported in wheat cultivars (Rao et al., 2025). By optimizing energy distribution and maintaining redox balance, HRW emerges as a promising strategy to improve crop resilience and productivity (Yu et al., 2023).

### 4.3 Influence of HRW treatment on TChl and Car in grape berry peels

Fruit ripening is characterized by chlorophyll degradation, Car accumulation, and cell wall breakdown. The transition from green to red coloration is primarily driven by the loss of chlorophyll and the biosynthesis and accumulation of pigments such as Car, TAC, and lycopene. These compounds contribute to the development of red, purple, or blue pigmentation, particularly in berries and grapes (Wu et al., 2020a; Francis and Abdulhameed, 2024). Our results showed no significant differences in TChl between HRW and control, both exhibiting a decrease in chlorophyll with ripening (**Figure 4A**), consistent with Fang et al. (2020), who found jasmonic acid upregulated chlorophyll degradation in broccoli. Thus, chlorophyll degradation may contribute to enhanced fruit coloration. ChlF, which reflect PSII efficiency and photoprotective capacity (Martínez-Lüscher et al., 2015), are closely linked to the balance between chlorophyll breakdown and Car synthesis during ripening. Declines in ChlF often coincide with structural changes in chloroplasts and the conversion to chromoplasts, where Car accumulate for photoprotection and antioxidant defense (Gambetta et al., 2021).

The results demonstrated that HRW treatment influenced the dynamics of Car accumulation in ‘Flame Seedless’ grapes. Car levels were lower in HRW-treated grapes compared to the control at DB44, DB54, and DB64 but surpassed the control at DB74, with both treatments showing a general decrease over time (**Figure 4B**). Car accumulation is regulated by redox balance, with ROS playing a key role in stimulating biosynthesis (Ali et al., 2024). Antioxidant enzymes help mitigate oxidative stress in berry tissues (Rashad et al., 2023), and their activity can indirectly modulate Car biosynthesis by controlling ROS levels that act as biosynthetic signals (El-Saadony et al., 2024).HRW-treated leaves exhibited higher antioxidant enzyme activity and lower levels of H_2_O_2_ and O_2_^·-^ on the final day of the experiment, indicating enhanced ROS scavenging capacity. The reduced ROS levels during early fruit development likely led to lower Car biosynthesis stimulation, explaining the reduced accumulation at earlier stages. In contrast, the prolonged effect of HRW may have slowed Car degradation in later stages, leading to higher accumulation at DB74. Similar findings in other fruits, such as tomatoes treated with hydrogen nanobubbles(HNW) (He et al., 2024), which indicated that HRW can enhance Car levels by preventing degradation.

### 4.4 Enhancement of TAC, TPC, TFC, and CIRG in berry peels by HRW treatment

Plants contain low-molecular-mass antioxidant metabolites such as TAC, TPC, and TFC, which play essential roles in protecting against, and scavenging ROS, and attracting pollinators (Anić et al., 2024). Anthocyanins represent the principal pigments that confer red and blue coloration in fruits and flowers. HRW has been reported to enhance anthocyanin accumulation in plants, such as radish (Zhang et al., 2018). In our study, HRW increased TAC in grape berry peels (**Figure 4C**), possibly by promoting the expression of key biosynthesis genes (Zhang et al., 2019). H_2_ mitigates oxidative stress by scavenging excessive ROS and maintaining cellular redox homeostasis (Liu et al., 2015), thereby preventing ROS-induced inhibition of phenylpropanoid and flavonoid biosynthetic enzymes such as PAL, CHS, and COMT (Liu et al., 2022). This redox balance allows efficient TAC production by sustaining the phenylpropanoid pathway and other secondary metabolite biosynthesis routes, leading to higher accumulation of phenolics, flavonoids, and anthocyanins (Liu et al., 2022). Moreover, H_2_ treatment integrates with other signaling molecules, including NO, H_2_S, MeJA, and ethylene, to fine-tune secondary metabolite production (Sana et al., 2025). Thus, the progressive increase in TAC during ripening is not only associated with pigmentation and protection but also reflects an improved metabolic environment resulting from ROS homeostasis and enhanced transcriptional activation of key biosynthetic genes (Liu et al., 2022; Sana et al., 2025). This pattern is consistent with findings in peach, where TAC accumulation occurs during ripening (Li et al., 2023). HRW-treated grapes exhibited higher TAC levels than controls across all ripening stages, likely due to a combination of factors: suppression of PPO activity, which minimizes oxidative degradation of TAC (Zhang et al., 2023a); enhanced protection against oxidative stress, which stabilizes TAC (Ortiz et al., 2024); and modulation of secondary metabolic pathways, leading to greater anthocyanin biosynthesis and retention, as observed in bananas and pakchoi (An et al., 2021; Siddiqui et al., 2022).

TPC and TFC are critical for mitigating oxidative stress and reducing oxidative damage in fruits and vegetables (Li et al., 2024a). Recent studies have demonstrated that H_2_ enhances TPC, TFC, and pigments in various fruits, including dried apple (Alwazeer, 2018), and strawberries (Alwazeer and Özkan, 2022). HRW treatment also alleviates oxidative damage by boosting TPC and TFC, thereby extending the shelf life of daylily buds (Hu et al., 2021) and lychee (Yun et al., 2021).These findings collectively highlight HRW’s potential in enhancing antioxidant capacity and preserving fruits quality.

In the present study on ‘Flame Seedless’ grapevines, TPC and TFC gradually declined during ripening (**Figures 4D, E**), consistent with the natural oxidative processes occurring in fruit tissues. Similar decreasing trends have been reported in grapevine (Anić et al., 2024), with further reductions in overripe stages attributed to enhanced climacteric respiration (Li et al., 2023). This reduction is primarily driven by enzymatic oxidation catalyzed by PPO, which converts phenolic compounds into oxidized derivatives, thereby lowering their detectable levels (Taghipour et al., 2024). Additionally, water accumulation in berries during ripening promotes the hydrolysis of high-molecular-weight phenolics (Jediyi et al., 2019), while the decline in primary metabolic activity limits the biosynthesis of these secondary metabolites (Li et al., 2023). Over time, polyphenols may also bind to other compounds, further decreasing total phenol content (Zhao et al., 2024). Environmental conditions, particularly temperature and light intensity, can also modulate phenolic biosynthesis and degradation (Chen et al., 2025).

Notably, HRW-treated grapes maintained higher TPC and TFC than the control throughout ripening, particularly in the later stages, due to both reduced PPO activity and increased activities of antioxidant enzymes (SOD, POD, and CAT). These changes enhanced ROS scavenging, thereby protecting phenolic compounds from oxidative damage and delaying their degradation. This trend aligns with previous observations in daylily buds (Hu et al., 2021), banana (Siddiqui et al., 2022), and pakchoi (An et al., 2021), where HRW mitigated phenolic loss by suppressing oxidative enzyme activity.

Color is a critical quality attribute that directly influences consumer preference and marketability of grapes. In the present study, HRW-treated grapes exhibit enhanced fruit coloration, as evidenced by increased CIRG values (**Figure 4F**), reflecting a more desirable visual phenotype. This enhancement is closely associated with the coordinated regulation of pigment metabolism, including chlorophyll degradation and the accumulation of Car and anthocyanins. Chlorophyll degradation is a hallmark of fruit ripening, marking the transition from green to mature coloration, whereas Car and anthocyanins contribute yellow, orange, and red hues, thereby reinforcing overall color development and providing additional antioxidant benefits (Olivares et al., 2017). HRW treatment simultaneously promotes carotenoid accumulation and supports anthocyanin biosynthesis. These effects collectively enhance fruit coloration during ripening, potentially providing growers with increased flexibility in harvest timing to better align with market demands (Karimi et al., 2022).Therefore, the observed improvements in color reflect an integrated modulation of pigment metabolism and antioxidant activity, emphasizing the role of HRW in optimizing both visual quality and nutritional value of grapes.

### 4.5 Influence of HRW on fruit firmness, TA, pH, total soluble sugars, and TSS in grape berries

Fruit quality is determined by multiple factors, including maturity, firmness, sweetness, pH, TSS, total soluble sugars, TA, and TAC. Firmness, a key indicator of fruit texture and consumer acceptability, typically decreases during ripening due to cell wall disassembly and enzymatic activity. In this study, ‘Flame Seedless’ grapes treated with HRW exhibited reduced firmness compared to the control at all sampling points (DB44, DB54, DB64, DB72) (**Figure 5A**). This observation aligns with previous reports indicating that HRW promotes fruit softening during pre-harvest stages by modulating cell wall components and enhancing the activity of cell wall-degrading enzymes (Yao et al., 2024). Conversely, postharvest studies have shown that HRW preserves firmness by inhibiting cell wall degradation, as observed in okra (Dong et al., 2023). These contrasting effects may be attributed to (1) the developmental stage of the fruit, (2) HRW’s role in preserving cell wall integrity postharvest, and (3) its stimulation of ethylene biosynthesis and activation of ripening-related genes during pre-harvest. Additionally, HRW’s antioxidant properties may influence oxidative processes differently depending on the timing of application (Yao et al., 2024). HRW concentration also plays a role, with higher concentrations accelerating firmness loss (Li et al., 2021). These findings underscore the complex role of HRW in fruit texture regulation and highlight the need for further investigation into its molecular mechanisms, particularly those related to developmental timing, oxidative modulation, and phytohormonal signaling.

Organic acids are central to plant metabolism and significantly influence fruit flavor, sweetness, and overall quality (Li et al., 2023). In our study, TA levels decreased progressively as grapes matured in both treatments, with HRW-treated grapes showing significantly lower TA values than the control (**Figure 5B**). This result is consistent with findings in cherry tomatoes, where HRW reduced TA content (Li et al., 2024a). As ripening progressed, pH values increased, with HRW-treated grapes consistently exhibiting higher pH than the control (**Figure 5C**). pH serves as an indicator of fruit quality and reflects metabolic differences between treatments (Zhong et al., 2023). The observed decrease in TA and increase in pH may be attributed to the degradation of organic acids through respiratory metabolism during ripening (Shi et al., 2025). Ripening is an energy-intensive process, relying on carbon sources such as sugars, amino acids, and organic acids (Taş et al., 2021). Given the low starch content in grapes, organic acids and cell wall components are the primary contributors to soluble sugar formation during maturation (Taş et al., 2021).

Total soluble sugars increased progressively throughout ripening in both treatments, with HRW-treated grapes consistently showing higher levels than the control (**Figure 5D**). Similar enhancements in sugar content have been reported in strawberries treated with (HNB) (Li et al., 2024a). This increase may be linked to HRW’s ability to modulate genes involved in sucrose and starch metabolism (Hou et al., 2021), as well as its positive effects on leaf physiology improving TChl, ChlF parameters, and antioxidant enzyme activity which collectively enhance photosynthetic efficiency and assimilate production (El-Saadony et al., 2024).

TA and TSS typically exhibit an inverse relationship during fruit ripening: as acidity decreases, TSS levels rise (Subedi et al., 2025). In grapes, TSS increased steadily from immature to mature stages in both treatments, peaking at DB74, with HRW-treated grapes consistently showing higher values than the control (**Figure 5E**). Similar trends have been observed in HRW-treated Hypsizygus marmoreus (Ali et al., 2024). This enhancement may be attributed to HRW’s antioxidant properties (He et al., 2024), as well as natural ripening processes such as water loss and the breakdown of complex carbohydrates into water-soluble sugars (Xiao et al., 2024).

### 4.6 Mechanisms underlying fruit quality improvements

HRW-mediated maintenance of PSII efficiency, reflected by Fv/Fm, ΦPSII, and qP, ensured effective photochemical energy conversion, minimized photoinhibition, and supported continuous carbohydrate production, contributing to both fruit growth and sugar accumulation (Choi et al., 2016). Additionally, efficient photosynthesis facilitated a balanced acid-to-sugar ratio, moderating fruit pH and TA, thereby enhancing flavor quality (Xu et al., 2022a; Du et al., 2024).Enhanced photosynthetic activity, together with improved antioxidant enzyme activities (SOD, POD, CAT), reduced ROS accumulation and oxidative degradation of pigments, preserving chlorophyll and Car and supporting biosynthesis and accumulation of phenolic compounds, flavonoids, and anthocyanins, which are key determinants of fruit color, nutritional value, and postharvest shelf-life (Arrones et al., 2022; Pérez-Labrada and Juárez-Maldonado, 2024). Moreover, stabilization of chloroplast ultrastructure and efficient energy distribution promoted sustained sugar transport to the fruit mesocarp, enhancing sweetness, and overall textural properties (Miyoshi et al., 2021; Xu et al., 2022a; Du et al., 2024).

**Figure 6:**
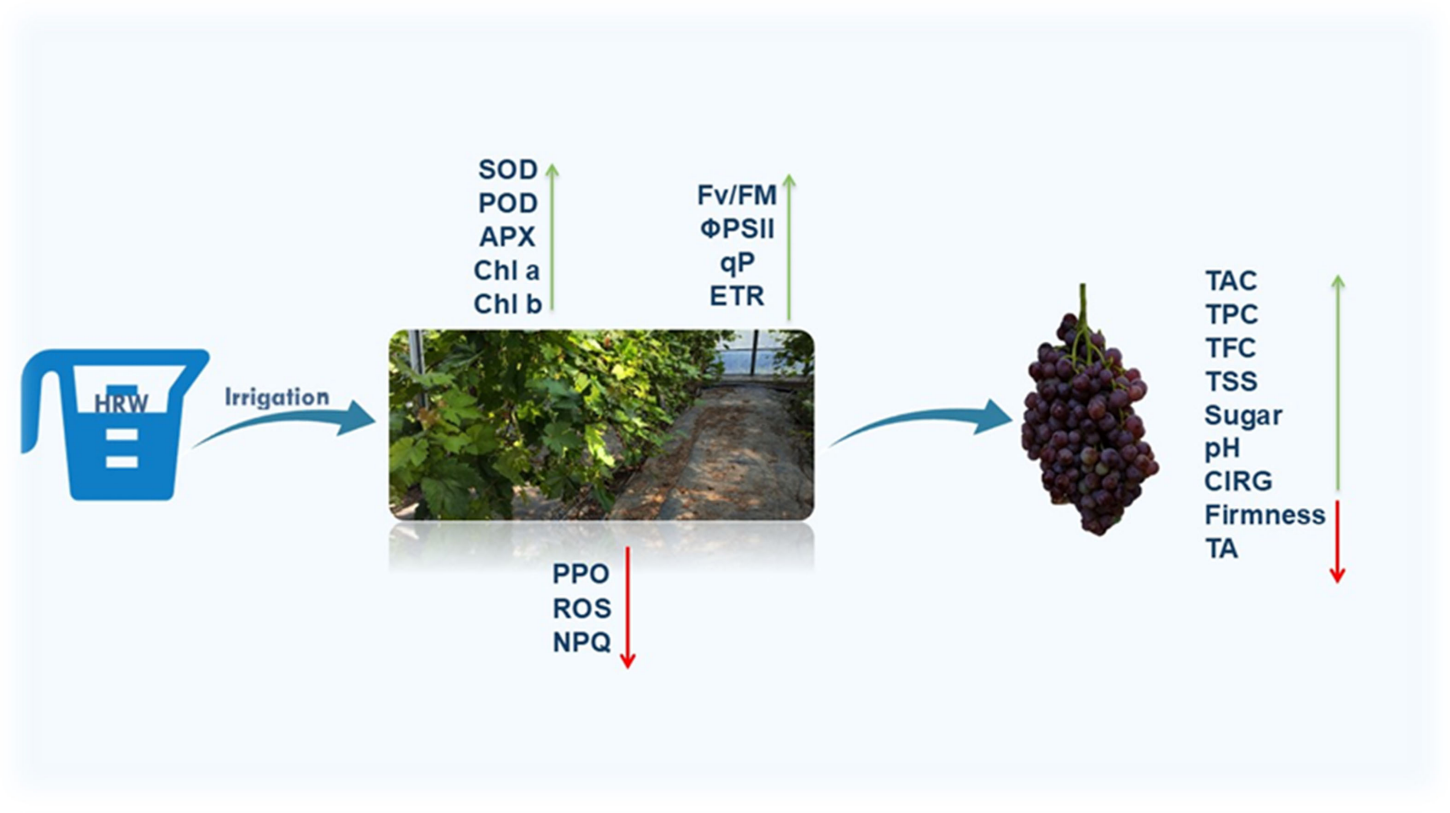
Proposed working model of HRW treatment in grape berry development. During berry development, HRW treatment enhanced antioxidant enzyme activities and reduced ROS levels, thereby fortifying grape leaves against oxidative stress. HRW treatment also improved photosynthetic efficiency by optimizing ChlF. Furthermore, HRW treatment promoted anthocyanin accumulation in berry peel, leading to improved fruit coloration and enhanced fruit quality by increasing total soluble solids and sugar content. HRW treatment also led to higher berry fresh weight and increased grape yield, suggesting a positive impact on resource allocation and fruit development. This suggests that HRW could lead to improvements in yield and fruit quality.

Overall, these results demonstrate that HRW-induced improvements in photosynthesis, ChlF, and antioxidant defense collectively enhance multiple fruit quality attributes, including TSS, soluble sugars, acidity, pH, chlorophyll and Car stability, TAC, TPC, TFC, firmness, berry and cluster weight, and yield, (**Figure 6**) providing a comprehensive mechanistic explanation for the observed benefits in ‘Flame Seedless’ grapes (Miyoshi et al., 2021; Arrones et al., 2022; Xu et al., 2022a; Du et al., 2024; Pérez-Labrada and Juárez-Maldonado, 2024; Yin et al., 2025).In summary, the improvements in fruit quality observed under HRW treatment can be mechanistically explained by enhanced photosynthetic efficiency, stabilized ChlF, and optimized pigment composition. Increased chlorophyll content in leaves and fruits improved light-harvesting capacity and carbon assimilation, thereby enhancing photosynthate availability for sugar accumulation, leading to higher TSS and increased sweetness in fruits.

## 5 Conclusion

HRW treatment demonstrates a multifaceted positive impact on ‘Flame Seedless’ grapes, enhancing their physiological and biochemical characteristics. The application of HRW significantly improved the antioxidant defense system in grape leaves by increasing the activity of key enzymes like SOD, POD, and CAT while reducing ROS accumulation. This, in turn, led to improved photosynthetic efficiency, as evidenced by higher chlorophyll content, enhanced ChlF parameters (Fv/Fm, ΦPSII, ETR, and qP), and reduced NPQ. Furthermore, HRW treatment positively influenced fruit quality by promoting TAC accumulation, modulating TPC and TFC, and improving the CIRG. The treatment also resulted in increased TSS and soluble sugar content, along with enhanced berry and cluster weights, ultimately leading to a higher estimated yield. These findings suggest that HRW treatment can be a valuable strategy for improving grape production by mitigating oxidative stress, enhancing photosynthetic performance, and improving overall fruit quality and yield. Further research at the molecular level is warranted to fully elucidate the underlying mechanisms of HRW’s beneficial effects.

## Data Availability

The original contributions presented in the study are included in the article/supplementary material. Further inquiries can be directed to the corresponding author.
